# Increased Rapid Eye Movement Sleep Is Associated With a Reduced Risk of Heart Failure in Middle-Aged and Older Adults

**DOI:** 10.3389/fcvm.2022.771280

**Published:** 2022-03-29

**Authors:** Binbin Zhao, Xiaoying Jin, Jian Yang, Qingyan Ma, Zai Yang, Wei Wang, Ling Bai, Xiancang Ma, Bin Yan

**Affiliations:** ^1^Department of Psychiatry, The First Affiliated Hospital of Xi’an Jiaotong University, Xi’an, China; ^2^Department of Clinical Research Center, The First Affiliated Hospital of Xi’an Jiaotong University, Xi’an, China; ^3^Department of Cardiology, The First Affiliated Hospital of Xi’an Jiaotong University, Xi’an, China

**Keywords:** percentage of REM sleep, total REM sleep time, sleep heart health study, polysomnography, heart failue

## Abstract

**Objectives:**

Rapid eye movement (REM) sleep is closely related to all-cause mortality. The aim of this study is to explore the role of REM sleep on the incident heart failure (HF).

**Methods:**

We selected 4490 participants (2480 women and 2010 men; mean age, 63.2 ± 11.0 years) from the Sleep Heart Health Study. HF was identified as the first occurrence during a mean follow-up period of 10.9 years. REM sleep including percentage of REM sleep and total REM sleep time were monitored using in-home polysomnography at baseline. Multivariable Cox regression analysis was utilized to explore the relationship between REM sleep and HF.

**Results:**

In total, 436 (9.7%) cases of HF were observed during the entire follow-up period. After adjusting for potential covariates, an increased percentage of REM sleep (per 5%) was independently associated with a reduced incidence of HF [hazard ratio (HR) 0.88, 95% confidence interval (CI) 0.82–0.94, *P* < 0.001]. A similar result was also found between total REM sleep time (increased per 5 min) and incident HF (HR 0.97, 95% CI 0.95–0.99, *P* < 0.001). Moreover, the fourth quartile of both percentage of REM sleep (HR 0.65, 95% CI 0.48–0.88, *P* = 0.005) and total REM sleep time (HR 0.64, 95% CI 0.45–0.90, *P* = 0.010) had lower risk of incident HF when compared with the first quartile.

**Conclusion:**

An increased percentage of REM sleep and total REM sleep time were associated with a reduced risk of HF. REM sleep may be a predictor of the incident HF.

**Clinical Trial Registration:**

[ClinicalTrials.gov], identifier [NCT00005275].

## Introduction

Sleep is an essential physiological phenomenon occurring in alternation with wakefulness, and good quality sleep is vital for maintaining health and homeostasis (1, 2). According to the World Health Organization, 27% of individuals worldwide suffer from sleep disorders. With the increasing pressure of work, study, and life, sleep issues have severely affected nearly 50–70 million people’s physical and mental health in the United States (3). There is growing evidence that poor sleep contributes to cardiovascular disease (CVD) and its risk factors, such as coronary artery disease, stroke, diabetes mellitus, hypertension, and obesity (4–8). However, the relationship between sleep and CVD deserves further investigation.

Rapid eye movement (REM) sleep and non-REM sleep are two critical stages of sleep. REM sleep, about 20–25% of total sleep time, is usually characterized by eye movement, increased heart rate and blood pressure, muscle relaxation, and low voltage and fast frequency in the electroencephalogram (9, 10). Recently, several studies have shown that individuals with a decreased percentage of REM sleep have high all-cause mortality (11, 12). Matthews et al. demonstrated that there was a close correlation between the percentage of REM sleep and sleep/wake ratios of blood pressure (13).

Despite the certainty of knowledge on the effects of REM sleep, the relationship between REM sleep and heart failure (HF) remains unclear. The purpose of this study was to explore whether there is an association between REM sleep (including percentage REM sleep and total REM sleep time) and incident HF based on the Sleep Heart Health Study (SHHS) datasets.

## Materials and Methods

### Study Population

Data were derived based on an existing dataset from the SHHS (ClinicalTrials.gov, identifier NCT00005275), which was a community-based multicenter prospective cohort study performed by the National Heart, Lung, and Blood Institute to investigate the cardiovascular and other consequences of sleep-disordered breathing between baseline (November 1, 1995 and January 31, 1998) and 2011. The detailed method and design of this study have been reported previously (14, 15). Briefly, a total of 6441 participants aged 40 years and older were recruited from several “parent” cohorts including the Atherosclerosis Risk in Communities study, the Cardiovascular Health Study, the Framingham Offspring and Omni study, the Strong Heart Study, the Health and Environment and Tucson Epidemiologic Study, and studies of hypertension in New York. All participants in the SHHS signed written consent, and the study protocol was approved by the institutional review board of each participating institution to collect sleep and questionnaire data. The data underlying this article are available in National Sleep Research Resource, at https://doi.org/10.25822/ghy8-ks59. The datasets were derived from sources in the public domain. Our access to the SHHS database was provided after acquiring a signed agreement with the Brigham and Women’s Hospital. The SHHS shared dataset from the National Sleep Research Resource does not include 637 individuals from the Strong Heart Study due to sovereignty issues. Exclusion criteria for our study were (1) previous CVD outcomes (*n* = 535); (2) participants who use CPAP or a mouthpiece (*n* = 7); and (3) no follow-up data (*n* = 772). Finally, 4490 participants were included in the present study (Supplementary Figure 1).

### Sleep Parameters

Polysomnography (PSG) is essential for the diagnosis and management of many sleep parameters. All individuals in the current study underwent electroencephalography-based overnight unattended PSG (P-Series, Compumedics, Abbotsville, VIC, Australia) at home (15). Supplementary Material showed the details regarding the specific technical aspects of the PSG measurement. The time and percentage of REM sleep were captured using PSG monitoring. In addition, the percentage of REM sleep was categorized into quartiles: Q1 (<15.8%; *n* = 1113), Q2 (15.8–20.1%; *n* = 1134), Q3 (20.2–24.0%; *n* = 1145), and Q4 (>24.0%; *n* = 1098). Moreover, total REM sleep time was also divided into quartiles: Q1 (<54.0 min; *n* = 1113), Q2 (54.0–73.5 min; *n* = 1162), Q3 (73.6–91.5 min; *n* = 1110), and Q4 (>91.5 min; *n* = 1105). Other sleep structure parameters, including time in stage 1 (min), time in stage 2 (min), time in stage 3 (min), percentage stage 1, percentage stage 2, and percentage stage 3, were also included in the current analysis. Sleep duration was defined as the total time in bed (Time from lights off to lights on, rounded to nearest minute) based on the PSG record. The apnea–hypopnea index (AHI) obtained from the PSG was identified as the number of apneas or hypopneas recorded during the study per hour of sleep, accompanied by at least a 4% drop in oxygen saturation (15).

### The Identification of Heart Failure

The criteria for HF was based on clinical signs and symptoms (such as rales, edema, dyspnea, and orthopnea), physiologic tests (decreased systolic function), and supportive findings (chest radiography or functional cardiac imaging), as previous study reported (16). HF was defined as the first occurrence during the average 10.9 years’ follow-up. The control group for HF patients was defined as participants who had no CVD outcomes at baseline and did not develop HF during the follow-up period when investigated the association between REM sleep and HF. Myocardial infarction (MI), stroke, and CVD death were also identified in the follow-up time. All the CVD events were evaluated in the parent cohorts using explicit protocols and identified for SHHS using follow-up interviews, written annual questionnaires, telephone contacts with study participants or next-of-kin, surveillance of local hospital records and community obituaries, and linkage with the Social Security Administration Death Master File (17).

### Covariates

Covariates including age, sex, race, education, marital status, smoking status, body mass index (BMI), alcohol use, caffeine use, benzodiazepine use, hypertension, diabetes mellitus, triglyceride, cholesterol and high-density lipoprotein (HDL) cholesterol, percent time below oxygen desaturation 90% (T90), and AHI were obtained from the SHHS baseline examination.

### Statistical Analyses

Continuous and categorical variables were summarized using Student’s *t*-test and Chi-squared tests, respectively. The results are presented as mean (±SD) and number (percentages). Moreover, unadjusted Kaplan–Meier survival curves were drawn to investigate the overall survival of individuals with different REM sleep quartiles. Cox regression analysis was used to calculate the hazard ratios (HRs) and 95% confidence intervals (CIs) in the relationship between REM sleep and HF. Multivariable Cox regression analysis adjusted for age, sex, race, education, marital status, smoking status, BMI, alcohol use, caffeine use, benzodiazepine use, hypertension, diabetes mellitus, triglyceride, cholesterol, HDL, sleep duration, T90, and AHI to examine the associations between REM sleep and HF. Subgroup analysis stratified by sex (men vs. women) and AHI (≥15 vs. <15 events/h) was performed when explore the role of REM sleep on the incidence of HF. All statistical analyses were conducted using SPSS (version 24.0; SPSS Inc.). A two-sided *P* < 0.05 was considered to be statistically significant.

## Results

### Study Population

Table 1 shows the study characteristics in participants with and without HF. The present study included 4490 individuals [2480 women (55.2%)] with a mean age of 63.8 ± 11.0 years. A total of 436 (9.7%) cases of HF was observed during the follow-up period. Individuals with HF were older and had more smokers, hypertension, diabetes mellitus than controls. In addition, HF patients had low level of percentage of REM sleep, total REM sleep time, T90, and AHI compared with controls.

**TABLE 1:** Study characteristics in participants with and without HF.

Characteristics	Total *n* = 4490	HF *n* = 436	Controls *n* = 4054	*P*-value
Age, years	63.2 ± 11.0	73.6 ± 7.8	62.1 ± 10.7	<0.001
Sex (%)				0.035
Men	2010 (44.8)	216 (49.5)	1794 (44.3)	
Women	2480 (55.2)	220 (50.5)	2260 (56.7)	
Race (%)				0.751
White	3904 (87.0)	377 (86.5)	3527 (87.0)	
Others	586 (13.0)	59 (13.5)	527 (13.0)	
Education (%)				<0.001
≤15 years	2564 (62.9)	316 (74.2)	2248 (61.6)	
>15 years	1511 (37.1)	110 (25.8)	1401 (38.4)	
Marry (%)				<0.001
Married	3548 (80.3)	314 (72.4)	3234 (81.2)	
Others	870 (19.7)	120 (27.6)	750 (18.8)	
Smoking status, *n* (%)				0.023
Current smoker	440 (9.9)	45 (10.4)	395 (9.8)	
Former smoker	1925 (43.0)	211 (48.6)	1714 (42.4)	
Never smoker	2112 (47.1)	178 (41.0)	1934 (47.8)	
BMI, kg/m^2^	28.3 ± 5.0	29.0 ± 4.9	28.2 ± 5.0	0.003
Hypertension, *n* (%)	1633 (36.4)	282 (64.7)	1351 (33.3)	<0.001
Diabetes mellitus, *n* (%)	286 (6.5)	79 (18.4)	207 (5.2)	<0.001
Alcohol use, *n* (%)				0.001
At least one drink per day	1838 (43.7)	154 (35.8)	1684 (44.6)	
None	2369 (56.3)	276 (64.2)	2093 (55.4)	
Caffeine use, *n* (%)				0.010
At least one intake per day	2749 (61.3)	242 (55.6)	2507 (62.0)	
None	1732 (38.7)	193 (44.4)	1539 (38.0)	
Benzodiazepine use, *n* (%)	241 (5.4)	33 (7.6)	208 (5.1)	0.030
Triglyceride, mL/dL	150.2 ± 99.4	155.7 ± 93.0	149.6 ± 100.1	0.231
Cholesterol, mL/dL	206.9 ± 38.1	204.6 ± 38.5	207.1 ± 38.0	0.194
HDL, mL/dL	51.1 ± 15.8	49.4 ± 13.8	51.3 ± 16.0	0.007
Sleep duration, h	7.3 ± 0.9	7.3 ± 1.0	7.3 ± 0.9	0.849
AHI, events/h	9.7 ± 13.0	12.1 ± 13.1	9.5 ± 13.0	<0.001
T90, %	3.3 ± 9.9	5.5 ± 13.5	3.0 ± 9.4	<0.001
**Sleep structure**				
REM sleep time (min)	72.4 ± 29.0	62.7 ± 28.3	73.4 ± 28.9	<0.001
Time in stage 1 (min)	18.7 ± 13.1	20.0 ± 15.0	18.6 ± 12.9	0.061
Time in stage 2 (min)	208.6 ± 56.8	205.3 ± 60.5	208.9 ± 56.4	0.204
Time in stage 3 (min)	65.2 ± 44.4	60.7 ± 47.4	65.7 ± 44.0	0.027
REM sleep time (%)	19.6 ± 6.7	17.7 ± 7.2	19.8 ± 6.7	<0.001
Time in stage 1 (%)	5.2 ± 3.8	5.8 ± 4.5	5.2 ± 3.8	0.004
Time in stage 2 (%)	57.3 ± 13.1	59.1 ± 14.1	57.1 ± 12.9	0.005
Time in stage 3 (%)	17.9 ± 11.9	17.3 ± 12.7	17.9 ± 11.8	0.348
Follow-up time, years	10.9 ± 2.8	9.4 ± 3.0	11.1 ± 2.7	<0.001

*AHI, apnea–hypopnea index; BMI, body mass index; HDL, high-density lipoprotein; HF, heart failure; REM, rapid eye movement; T90, percent time below oxygen desaturation 90%. Results are presented as mean ± SD or number (percentage). The P-values represent the difference between two groups.*

### The Relationship Between Rapid Eye Movement Sleep and Incident Heart Failure

**FIGURE 1:**
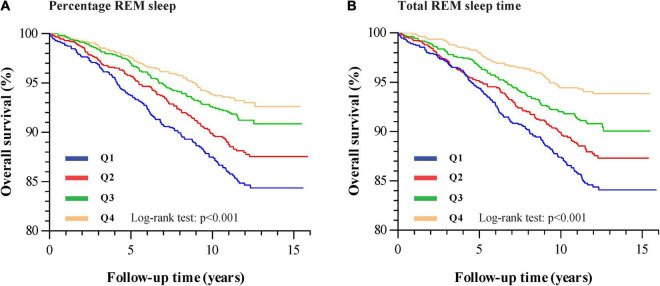
Kaplan–Meier plots of overall survival for HF stratified by percentage REM sleep quartiles [**(A)** Q1: <15.8%; Q2: 15.8–20.1%; Q3: 20.2–24.0%; Q4: >24.0%] and total REM sleep time quartiles [**(B)** Q1: <54.0 min; Q2: 54.0–73.5 min; Q3: 73.6–91.5 min; Q4: >91.5 min].

Unadjusted Kaplan–Meier survival curves showed that HF event rates increased with a decrease in REM sleep percentage (Figure 1A; Log-rank test: *P* < 0.001). After adjusting for age, sex, race, education, marital status, smoking status, BMI, alcohol use, caffeine use, benzodiazepine use, hypertension, diabetes mellitus, triglyceride, cholesterol, HDL, sleep duration, T90, and AHI (natural log-transformed), multivariable Cox regression analysis showed that an elevated percentage of REM sleep (per 5%) was significantly associated with a reduced risk of HF (HR 0.88, 95% CI 0.82–0.94, *P* < 0.001). Moreover, individuals in the fourth quartile of percentage REM sleep had a significantly lower risk of HF than those in the first quartile (HR 0.65, 95% CI 0.48–0.88, *P* = 0.005) (Table 2).

**TABLE 2:** Hazard ratios and 95% CIs for REM sleep associated with incident HF.

REM sleep traits	Persons (*n*)	Event, *n* (%)	Univariable model		Age and gender adjusted		Multivariable adjusted	
					
Percentage REM sleep	4490	436 (9.7)	HR (95% CI)	*P*	HR (95% CI)	*P*	HR (95% CI)	*P*
Q4 (>24.0%)	1098	148 (13.5)	0.44 (0.33–0.58)	<0.001	0.57 (0.43–0.76)	<0.001	0.65 (0.48–0.88)	0.005
Q3 (20.2–24.0%)	1145	126 (11.0)	0.55 (0.43–0.72)	<0.001	0.67 (0.51–0.86)	0.002	0.78 (0.59–1.04)	0.087
Q2 (15.8–20.1%)	1134	91 (8.0)	0.78 (0.61–0.98)	0.036	0.87 (0.69–1.10)	0.253	1.07 (0.84–1.38)	0.577
Q1 (<15.8%)	1113	71 (6.4)	1 (Ref)		1 (Ref)		1 (Ref)	
Continuous (per 5%)			0.80 (0.75–0.85)	<0.001	0.85 (0.80–0.91)	<0.001	0.88 (0.82–0.94)	<0.001
Total REM sleep time	4490	436 (9.7)	HR (95% CI)	*P*	HR (95% CI)	*P*	HR (95% CI)	*P*
Q4 (>91.5 min)	1105	149 (13.5)	0.37 (0.28–0.50)	<0.001	0.56 (0.42–0.76)	<0.001	0.64 (0.45–0.90)	0.010
Q3 (73.6–91.5 min)	1110	130 (11.7)	0.59 (0.46–0.76)	<0.001	0.71 (0.54–0.91)	0.008	0.79 (0.59–1.06)	0.107
Q2 (54.0–73.5 min)	1162	95 (8.2)	0.78 (0.62–0.99)	0.041	0.85 (0.67–1.08)	0.179	0.94 (0.73–1.21)	0.611
Q1 (<54.0 min)	1113	62 (5.6)	1 (Ref)		1 (Ref)		1 (Ref)	
Continuous (per 5 min)			0.94 (0.92–0.95)	<0.001	0.96 (0.94–0.97)	<0.001	0.97 (0.95–0.99)	<0.001

*95% CI, 95% confidence interval; HF, heart failure; HR, hazard ratio; REM, rapid eye movement sleep. Percentage REM sleep quartiles (Q1: <15.8%; Q2: 15.8–20.1%; Q3: 20.2–24.0%; Q4: >24.0%). Total REM sleep time quartiles (Q1: <54.0 min; Q2: 54.0–73.5 min; Q3: 73.6–91.5 min; Q4: >91.5 min). The comparison was made in the participants with and without HF. Ref is referred to the first quantile of percentage REM sleep or total REM sleep time. Multivariable Cox regression analysis adjusted by age, sex, race, education, marital status, smoking status, BMI, alcohol use, caffeine use, benzodiazepine use, hypertension, diabetes mellitus, triglyceride, cholesterol, HDL, sleep duration, T90, and AHI (natural log-transformed).*

We also explored the association between total REM sleep time and HF. Similar to the results for REM sleep percentage, total REM sleep time (increased per 5 min) was found to be independently associated with HF (HR 0.97, 95% CI 0.95–0.99, *P* < 0.001). In addition, individuals in the fourth quartile of the total REM sleep time had the highest overall survival rate (Figure 1B; Log-rank test: *P* < 0.001). Total REM sleep time within the fourth quartile was associated with a lower risk of HF (HR 0.64, 95% CI 0.45–0.90, *P* = 0.010) than in the first quartile (Table 2). We also showed the results of final fully adjusted model in our Supplementary Tables 1, 2.

### The Association of Rapid Eye Movement Sleep With Myocardial Infarction, Stroke, and Cardiovascular Disease Death

During the follow-up time, 282 cases of MI, 201 cases of stroke, and 238 cases of CVD death occurred. We also explored role of percentage REM sleep and total REM sleep time on the incidence of MI, stroke, and CVD death. Both percentage REM sleep (HR 0.90, 95% CI 0.81–0.99, *P* = 0.032) and total REM sleep time (HR 0.97, 95% CI 0.94–0.99, *P* = 0.010) were associated with decreased risk of CVD death, but not associated with MI and stroke (Table 3).

**TABLE 3:** Hazard ratios and 95% CIs for REM sleep associated with MI, stroke, and CVD death.

			REM sleep quartiles				
REM traits	CVD events	Event, *n* (%)	Q1 (low)	Q2	Q3	Q4 (high)	Overall trend (per 5 unit)
Percentage REM sleep (%)	MI	282 (6.3)	1 (Ref)	1.03 (0.72–1.46)	0.98 (0.68–1.41)	0.98 (0.67–1.41)	1.01 (0.92–1.11)
	Stroke	201 (4.5)	1 (Ref)	0.86 (0.58–1.29)	0.72 (0.47–1.11)	0.90 (0.60–1.36)	0.95 (0.85–1.05)
	CVD death	238 (5.3)	1 (Ref)	0.75 (0.52–1.08)	0.72 (0.49–1.07)	0.74 (0.50–1.09)	0.90 (0.81–0.99) *
Total REM sleep time (min)	MI	282 (6.3)	1 (Ref)	0.93 (0.65–1.33)	1.05 (0.72–1.52)	1.09 (0.71–1.65)	1.00 (0.98–1.03)
	Stroke	201 (4.5)	1 (Ref)	0.75 (0.50–1.12)	0.78 (0.51–1.21)	0.75 (0.46–1.22)	0.98 (0.96–1.01)
	CVD death	238 (5.3)	1 (Ref)	0.78 (0.54–1.12)	0.74 (0.50–1.10)	0.71 (0.45–1.11)	0.97 (0.94–0.99)^#^

*95% CI, 95% confidence interval; CVD, cardiovascular disease; HR, hazard ratio; MI, myocardial infarction; REM, rapid eye movement sleep. Percentage REM sleep quartiles (Q1: <15.8%; Q2: 15.8–20.1%; Q3: 20.2–24.0%; Q4: >24.0%). Total REM sleep time quartiles (Q1: <54.0 min; Q2: 54.0–73.5 min; Q3: 73.6–91.5 min; Q4: >91.5 min). The comparison was made in the participants with and without individual CVD events (MI, stroke, and CVD death), respectively. Ref is referred to the first quantile of percentage REM sleep or total REM sleep time. Multivariable Cox regression analysis adjusted by age, sex, race, education, marital status, smoking status, BMI, alcohol use, caffeine use, benzodiazepine use, hypertension, diabetes mellitus, triglyceride, cholesterol, HDL, sleep duration, T90, and AHI (natural log-transformed).*

*P < 0.05; ^#^P < 0.01.

### Other Sleep Structure Parameters and Cardiovascular Disease Events

We also investigated the role of sleep characteristics (including time in stage 1, time in stage 2, time in stage 3, percentage stage 1, percentage stage 2, and percentage stage 1) on the incidence of HF, MI, stroke, and CVD death. No significant association was found after adjusting for potential confounding variables (Supplementary Table 3).

### Subgroup Analysis

We further conducted subgroup analysis stratified by AHI ≥ 15 and AHI < 15 events/h and sex (men vs. women) to further explore the role of REM sleep percentage and total REM sleep time on the incidence of HF. The results showed that both percentage and total time of REM sleep were still associated with incident HF in these subgroup analyses (Supplementary Tables 4, 5). Moreover, no significant interaction was found in these analyses (all the *P*_*interaction*_ > 0.05).

## Discussion

In the present study, we utilized a large-scale community-based population from SHHS to investigate the association between REM sleep and incidence of HF. Our study was a cohort-study design and the comparison of percentage REM sleep and total REM sleep time was made in the individuals with and without HF. REM sleep traits including percentage REM sleep and total REM sleep time were monitored by over-night PSG at home. Our multivariable Cox regression analysis demonstrated that middle-aged and older adults with an elevated percentage of REM sleep and total REM sleep time had a reduced risk of incident HF.

Poor lifestyles and behaviors are considered the main causes of negative cardiovascular outcomes (18). Sleep, a basic human behavior, is believed to be related to the risk of CVD and other health outcomes (19, 20). REM sleep is an important aspect of human sleep and often accompanied with vivid dreaming and high level of brain activity, which also has substantial effects on the physiological functions of the individual (21, 22). Previous studies showed that participants with a depressed mood spent less time in REM sleep (23). Besides, decreased REM sleep time in individuals is found to be closely related to worsening cognitive performance (24, 25). Matthews et al. also demonstrated a significant correlation between a lower proportion of REM sleep and a greater sleep/wake ratio of blood pressure (13). Increasing evidences have shown that REM sleep is vital to human health, but there was no evidence regarding the role of REM sleep on the incident HF. In this study, we provided evidences that both increased percentage REM sleep and total REM sleep time were significantly associated with low risk of incident HF. Sleep disordered breathing (SDB) is characterized by abnormal respiration during sleep and may influence the sleep continuity (26). SDB was also closely related to an increased risk of incident HF (27). Azarbarzin et al. found that sleep apnea-specific hypoxic burden was associated with the HF risk (28). We therefore adjusted AHI and T90 in our multivariable Cox regression analysis. Moreover, we performed subgroup analysis stratified by SDB severity (AHI ≥ 15 vs. AHI < 15 events/h) to examine whether SDB was potential confounders in the relationship between REM sleep and HF. The results revealed that percentage REM sleep and total REM sleep time were still associated with incidence of HF in subgroup analysis. Our findings indicated that REM sleep traits including percentage REM sleep and total REM sleep time might be marker to predict incident HF.

Previous studies showed that REM sleep is associated with all-cause mortality (11, 12). A decreased percentage of REM sleep was found to be associated with high CVD mortality in the Outcomes of Sleep Disorders in Older Men (MrOS) Sleep Study Cohort, but this relationship was not found in the Wisconsin Sleep Cohort (12). We also explored the role of REM sleep on the MI, stroke, and CVD death. Our results found a significant association of both percentage of REM sleep and total REM sleep time with CVD death based on SHHS, which could support the results of MrOS. However, no significant association was found between REM sleep and incidence of MI and stroke.

Previous studies revealed that the initiation and maintenance of REM sleep was related to the brainstem, forebrain, and hypothalamus (29). Gonnissen et al. showed that the changes of REM sleep time could be caused by circadian misalignment. Besides, reduced REM sleep may be associated with HPA-axis dysregulation and decreased insulin sensitivity (30). Additionally, decreased REM sleep was also found to have a high cortisol concentration (31). We speculate that the abnormal circadian rhythm and neuroendocrine function may contribute to the increased risk of CHF. The underlying biological mechanisms of REM sleep leading to an increased risk of HF still deserved further investigation.

The current study has some strengths. To our knowledge, it is the first to investigate the effect of REM sleep on the incidence of HF. REM sleep, including percentage and total time, was objectively monitored using PSG records, and our findings were based on a large community-based population. Nevertheless, this study also has several limitations. The objects of our analysis were mostly middle-aged and older Caucasian adults; therefore, the generalization of our conclusions to young people and other races merits careful consideration. Second, objective REM sleep was evaluated using a single-night PSG and may not fully reflect the significant value of REM sleep. Multiple long-term PSG monitoring may provide more accurate sleep parameters. Third, several parent cohorts oversampled snorers to increase the study-wide prevalence of SDB in SHHS. Therefore, our study population could not represent the general community population. Finally, we lack of data such as B-type natriuretic peptide (BNP), kidney function and left ventricular parameters such as ejection fraction, end-diastolic volume and end-systolic volume in the SHHS database that is closely related to the HF. We will investigate the effect of REM sleep on the changes of BNP, renal function and left ventricular function in our following study.

## Conclusion

Our study provides evidence that increased percentage and total time of REM sleep were associated with a decreased risk of incident HF in middle-aged and older adults. Percentage REM sleep and total REM sleep time may be predictors for the incidence of HF. Monitoring REM sleep to fully understand the nocturnal autonomic nervous activity of people may contribute to prevent HF in the early stages.

## Data Availability Statement

The datasets presented in this study can be found in online repositories. The names of the repository/repositories and accession number(s) can be found below: https://doi.org/10.25822/ghy8-ks59.

## Ethics Statement

The studies involving human participants were reviewed and approved by the Boston University, Case Western Reserve University, Johns Hopkins University, Missouri Breaks Research, Inc., New York University Medical Center, University of Arizona, University of California at Davis, University of Minnesota – Clinical and Translational Science Institute, and University of Washington. The patients/participants provided their written informed consent to participate in this study.

## Author Contributions

BY and XM raised the idea for the study and handled the supervision in our study. BZ, XJ, JY, QM, LB, ZY, and WW contributed to the study design, writing, and review of the report. BY acquired the data in SHHS and participated in further data analysis. All authors approved the final version of the report.

## Conflict of Interest

The authors declare that the research was conducted in the absence of any commercial or financial relationships that could be construed as a potential conflict of interest.

## Publisher’s Note

All claims expressed in this article are solely those of the authors and do not necessarily represent those of their affiliated organizations, or those of the publisher, the editors and the reviewers. Any product that may be evaluated in this article, or claim that may be made by its manufacturer, is not guaranteed or endorsed by the publisher.

## Abbreviations

AHI: apnea–hypopnea index
BMI: body mass index
HF: heart failure
CI: confidence interval
CVD: cardiovascular disease
HDL: high-density lipoprotein
HR: hazard ratio
MI: myocardial infarction
MrOS: Outcomes of Sleep Disorders in Older Men
PSG: polysomnography
REM: rapid eye movement
SHHS: Sleep Heart Health Study
T90: percent time below oxygen desaturation 90%.
