# Supplementary material for: Increased Rapid Eye Movement Sleep Is Associated With a Reduced Risk of Heart Failure in Middle-Aged and Older Adults
Source: Front Cardiovasc Med. 2022 Mar 29;9:771280. doi: 10.3389/fcvm.2022.771280 (PMC9001949; doi:10.3389/fcvm.2022.771280)
Supplement: Supplementary file 1 [file Data_Sheet_1.docx]

**Supplementary methods**

All the participants in the Sleep Heart Health Study (SHHS) underwent in-home polysomnography (PSG) to collect data of sleep parameters. Percentage of REM sleep and total REM sleep time were obtained from PSG records. The Compumedics sleep monitor was set up by a team of two individuals (one sleep technician and one helper) who have been specifically trained and certified. The sleep monitor is battery operated so the participant is not potentially in connection with electrical outlets. PSG records were obtained in an unattended setting. The next morning a technician returned to the participant’s home at a pre-arranged time to collect the sleep monitor and self-administered surveys. The PSG data were then sent to the Reading Center for processing and the paper forms submitted for local data entry. If the study was inadequate, a repeat study request is initiated. The entire monitoring process tried to make participants consistent with their usual sleep.

**Technical Notes on SHHS1**

The Compumedics P-Series Sleep Monitoring System used for SHHS1 consisted of a Main Unit and Patient Interface Box (PIB). The signals were obtained by the use of electrodes/sensors that connected to the PIB via Harwin connectors. The PIB connected to the Main Unit using an analogue cable and signals were recorded in digitized format onto a solid-state memory device (PCMCIA Memory Card) in the Main Unit. The montage was set up so that only 2.01 Mb/hour of data was recorded, providing 10 hours of data for the 20 Mb flashcards which were used. The PIB had 6 switch banks with toggle switches which would control the amplifiers (on/off). The main unit was powered by a rechargeable Nickel Metal Hydride battery. The PIB and loose electrode wires and sensor cables were supported by a cloth “bib/vest” with the recording unit placed in the center pocket. This bib/vest was placed over the participants’ nightclothes. After collection the study would be downloaded to a computer via serial lead, battery recharged and flash card reformatted.

The recording montage consisted of : (1) C3/A2 and C4/A1 EEGs, sampled at 125 Hz; (2) right and left electrooculograms (EOGs), sampled at 50 Hz; (3) a bipolar submental electromyogram (EMG), sampled at 125 Hz; (4) thoracic and abdominal excursions (THOR and ABDO), recorded by inductive plethysmography bands and sampled at 10 Hz; (5) “airflow” detected by a nasal-oral thermocouple (Protec, Woodinville, WA), sampled at 10 Hz; (6) finger-tip pulse oximetry (Nonin, Minneapolis, MN) sampled at 1 Hz; (7) ECG from a bipolar lead, sampled at 125 Hz for most SHHS-1 studies and 250 Hz for SHHS-2 studies; (8) Heart rate (PR) derived from the ECG and sampled at 1 Hz; (9) body position (using a mercury gauge sensor) ambient light (on/off, by a light sensor secured to the recording garment). The details about montage and sampling rate information on SHHS1 is below:

| **Channel** | **EDF Label** | **Input 1** | **Input 2** | **Sampling rate (Hz)** | **Hardware filters (Hz)** | **Sensor type** |
| --- | --- | --- | --- | --- | --- | --- |
| SpO2 | SaO2 | SaO2 | - | 1 | - | Nonin XPOD 3011, 8000 sensor |
| Heart Rate | H.R. | H.R. | - | 1 | - | Nonin XPOD 3011, 8000 sensor |
| Linked EEG | EEG (sec) | C3 | A2 | 125 | High pass 0.15 | Gold cup electrode |
| Linked EEG | EEG | C4 | A1 | 125 | High pass 0.15 | Gold cup electrode |
| ECG | ECG | ECG | - | 125 | High pass 0.15 | Ag/AgCl patch |
| Left EOG | EOG(L) | EOG(L) | PG1 | 50 | High pass 0.15 | Gold cup electrode |
| Right EOG | EOG(R) | EOG(R) | PG1 | 50 | High pass 0.15 | Gold cup electrode |
| EMG | EMG | EMG | - | 125 | High pass 0.15 | Gold cup electrode |
| Thorax | Thor RES | Thor RES | - | 10 | High pass 0.05 | Respitrace Inductance Plethysmography |
| Abdomen | Abdo RES | Abdo RES | - | 10 | High pass 0.05 | Respitrace Inducatnce Plethysmography |
| Position | Position | Position | - | 1 | High pass 0.05 | Internal mercury gauge |
| Light | Light | Light | - | 1 | - | External ambient light sensor |
| Airflow | New Air | New Air | - | 10 | High pass 0.05 | ProTech thermistor M325 (New Air channel 17) |
| Airflow (alternate) | Airflow | Airflow | - | 10 | High pass 0.05 | Compumedics thermistor (AUX channel 12) |
| Oximetry Status | OX STAT | OX STAT | - | 1 | - | Nonin XPOD 3011, 8000 sensor |
| REF | - | Fpz | - | - | - | - |
| Ground | - | PG1 | - | - | - | - |
