# Supplementary material for: Increased Rapid Eye Movement Sleep Is Associated With a Reduced Risk of Heart Failure in Middle-Aged and Older Adults
Source: Front Cardiovasc Med. 2022 Mar 29;9:771280. doi: 10.3389/fcvm.2022.771280 (PMC9001949; doi:10.3389/fcvm.2022.771280)
Supplement: Supplementary file 2 [file Table_1.DOCX]

Supplement Table 1 HRs and 95% CIs for Percentage REM sleep associated with incident HF

|  | Multivariable adjusted^a^ | |
| --- | --- | --- |
| All subjects | HR (95%CI) | P |
| Percentage REM sleep (per 5 %) | 0.88 (0.82-0.94) | <0.001 |
| Age | 1.13 (1.11-1.14) | <0.001 |
| Gender |  |  |
| Male | 1.27 (1.00-1.63) | 0.055 |
| Female | 1 |  |
| Race |  |  |
| White | 0.85 (0.61-1.19) | 0.337 |
| Other | 1 |  |
| Education |  |  |
| ≤15 years | 1.35 (1.07-1.70) | 0.010 |
| >15 years | 1 |  |
| Marital Status |  |  |
| Married | 0.65 (0.51-0.82) | <0.001 |
| Other | 1 |  |
| BMI |  |  |
| ≥30 | 1.27 (0.95-1.69) | 0.104 |
| 25.0-29.9 | 1.04 (0.79-1.37) | 0.770 |
| 18.5-24.9 | 1 |  |
| Smoking status |  |  |
| Current smoker | 1.71 (1.19-2.45) | 0.004 |
| Former smoker | 1.31 (1.05-1.63) | 0.016 |
| Never smoker | 1 |  |
| Alcohol use |  |  |
| At least one drink per day | 0.84 (0.68-1.05) | 0.135 |
| None | 1 |  |
| Caffeine use |  |  |
| At least one intake per day | 0.82 (0.67-1.01) | 0.066 |
| None | 1 |  |
| Benzodiazepine use |  |  |
| Yes | 1.10 (0.76-1.60) | 0.613 |
| No | 1 |  |
| Diabetes mellitus |  |  |
| Yes | 2.09 (1.60-2.72) | <0.001 |
| No | 1 |  |
| Hypertension |  |  |
| Yes | 2.06 (1.67-2.55) | <0.001 |
| No | 1 |  |
| Triglycerides | 1.00 (1.00-1.00) | 0.577 |
| Cholesterol | 0.99 (0.99-1.00) | 0.021 |
| HDL Cholesterol | 1.00 (0.99-1.01) | 0.820 |
| Sleep duration |  |  |
| <6h | 1.05 (0.85-1.28) | 0.670 |
| >8h | 1.01 (0.25-4.09) | 0.990 |
| 6-8 h | 1 |  |
| T90 | 1.01 (1.00-1.02) | 0.003 |
| AHI | 0.99 (0.99-1.00) | 0.102 |

AHI, apnea hypopnea index; BMI, body mass index; HDL, high-density lipoprotein; HF, heart failure; REM, rapid eye movement; T90, percent time below oxygen desaturation 90%.

AHI was natural log-transformed.

Supplement Table 2 HRs and 95% CIs for total REM sleep time associated with incident HF

|  | Multivariable adjusted^a^ | |
| --- | --- | --- |
| All subjects | HR (95%CI) | P |
| Total REM sleep time (per 5 min) | 0.97 (0.95-0.99) | <0.001 |
| Age | 1.13 (1.11-1.14) |  |
| Gender |  |  |
| Male | 1.27 (0.99-1.62) | 0.059 |
| Female | 1 |  |
| Race |  |  |
| White | 0.85 (0.61-1.19) | 0.338 |
| Other | 1 |  |
| Education |  |  |
| ≤15 years | 1.35 (1.07-1.69) | 0.011 |
| >15 years | 1 |  |
| Marital Status |  |  |
| Married | 0.65 (0.51-0.82) | <0.001 |
| Other | 1 |  |
| BMI |  |  |
| ≥30 | 1.26 (0.94-1.67) | 0.121 |
| 25.0-29.9 | 1.04 (0.79-1.36) | 0.799 |
| 18.5-24.9 | 1 |  |
| Smoking status |  |  |
| Current smoker | 1.70 (1.19-2.44) | 0.004 |
| Former smoker | 1.31 (1.05-1.63) | 0.017 |
| Never smoker | 1 |  |
| Alcohol use |  |  |
| At least one drink per day | 0.84 (0.67-1.01) | 0.064 |
| None | 1 |  |
| Caffeine use |  |  |
| At least one intake per day | 0.82 (0.67-1.01) | 0.064 |
| None | 1 |  |
| Benzodiazepine use |  |  |
| Yes | 1.10 (0.75-1.60) | 0.627 |
| No | 1 |  |
| Diabetes mellitus |  |  |
| Yes | 2.09 (1.60-2.73) | <0.001 |
| No | 1 |  |
| Hypertension |  |  |
| Yes | 2.06 (1.66-2.55) | <0.001 |
| No | 1 |  |
| Triglycerides | 1.00 (1.00-1.00) | 0.606 |
| Cholesterol | 0.99 (0.99-1.00) | 0.022 |
| HDL Cholesterol | 1.00 (0.99-1.01) | 0.787 |
| Sleep duration |  |  |
| <6h | 0.93 (0.75-1.16) | 0.511 |
| >8h | 1.01 (0.27-4.48) | 0.890 |
| 6-8 h | 1 |  |
| T90 | 1.01 (1.00-1.02) | 0.003 |
| AHI | 0.99 (0.99-1.00) | 0.102 |

AHI, apnea hypopnea index; BMI, body mass index; HDL, high-density lipoprotein; HF, heart failure; REM, rapid eye movement; T90, percent time below oxygen desaturation 90%.

AHI was natural log-transformed.

Supplement Table 3 HRs and 95% CIs for other sleep structure parameters associated with CVD events

| **CVD events** | Sleep structure | | | | | |
| --- | --- | --- | --- | --- | --- | --- |
|  | Time in Stage 1 | Time in Stage 2 | Time in Stage 3 | Percentage stage 1 | Percentage stage 2 | Percentage stage 3 |
| HF |  |  |  |  |  |  |
| Univariate models | 1.01 (1.00-1.02)* | 1.00 (1.00-1.00) | 1.00 (1.00-1.00) | 1.04 (1.02-1.07)& | 1.01 (1.01-1.02)& | 1.00 (0.99-1.00) |
| Age and gender adjusted | 1.00 (1.00-1.01) | 1.00 (1.00-1.00) | 1.00 (1.00-1.00) | 1.02 (1.00-1.05) | 1.01 (1.00-1.01) | 1.00 (0.99-1.01) |
| Multivariable adjusted^a^ | 1.00 (0.99-1.01) | 1.00 (1.00-1.00) | 1.00 (1.00-1.00) | 1.00 (0.98-1.03) | 1.00 (1.00-1.01) | 1.01 (1.00-1.01) |
| MI |  |  |  |  |  |  |
| Univariate models | 1.01 (1.00-1.02) | 1.00 (1.00-1.00) | 1.00 (0.99-1.00)# | 1.03 (1.00-1.06) | 1.01 (1.00-1.02) | 0.99 (0.99-1.00)* |
| Age and gender adjusted | 1.00 (0.99-1.01) | 1.00 (1.00-1.00) | 1.00 (1.00-1.00) | 0.99 (0.96-1.02) | 1.00 (0.99-1.01) | 1.00 (1.00-1.01) |
| Multivariable adjusted^a^ | 1.00 (0.99-1.01) | 1.00 (1.00-1.00) | 1.00 (1.00-1.00) | 0.98 (0.95-1.02) | 1.00 (0.99-1.01) | 1.00 (0.99-1.02) |
| Stroke |  |  |  |  |  |  |
| Univariate models | 1.00 (0.99-1.01) | 1.00 (1.00-1.00) | 1.00 (1.00-1.00) | 1.00 (0.96-1.03) | 1.01 (1.00-1.02) | 1.00 (1.00-1.01) |
| Age and gender adjusted | 1.00 (0.99-1.01) | 1.00 (1.00-1.00) | 1.00 (1.00-1.00) | 1.25 (0.93-1.67) | 1.01 (1.00-1.02) | 1.00 (0.99-1.01) |
| Multivariable adjusted^a^ | 0.99 (0.98-1.01) | 1.00 (1.00-1.00) | 1.00 (1.00-1.00) | 0.97 (0.93-1.01) | 1.01 (1.00-1.02) | 1.00 (0.99-1.01) |
| CVD death |  |  |  |  |  |  |
| Univariate models | 1.00 (0.99-1.01) | 1.00 (1.00-1.00) | 1.00 (0.99-1.00) | 1.02 (0.99-1.06) | 1.01 (1.01-1.02) # | 1.00 (0.98-1.01) |
| Age and gender adjusted | 1.00 (0.99-1.01) | 1.00 (1.00-1.00) | 1.00 (1.00-1.00) | 1.00 (0.97-1.04) | 1.01 (1.00-1.02) | 1.00 (0.99-1.01) |
| Multivariable adjusted^a^ | 0.99 (0.98-1.00) | 1.00 (1.00-1.00) | 1.00 (1.00-1.00) | 0.98 (0.95-1.02) | 1.01 (1.00-1.02) | 1.00 (0.99-1.01) |

95% CI, 95% confidence interval; CVD, cardiovascular disease; HF, heart failure; HR, hazard ratio; MI, myocardial infarction; REM, rapid eye movement sleep; T90, percent time below oxygen desaturation 90%.

a adjusted by age, sex, race, education, marital status, smoking status, BMI, alcohol use, caffeine use, benzodiazepine use, hypertension, diabetes mellitus, triglyceride, cholesterol, HDL, sleep duration, T90 and AHI (natural log-transformed)

P<0.05 *; P<0.01 #; P<0.001 &

Supplement Table 4 Multivariable Cox regression analysis for percentage REM sleep and total REM sleep time associated with CVD events stratified by sex.

|  |  | Men  (n=2011) | | Women  (n=2481) | |  |
| --- | --- | --- | --- | --- | --- | --- |
| REM traits | CVD events | HR (95%CI) | P | HR (95%CI) | P | P_interaction_ |
| Percentage REM sleep (per 5 %) | HF | 0.87 (0.78-0.97) | 0.011 | 0.89 (0.81-0.97) | 0.012 | 0.870 |
|  | MI | 1.07 (0.94-1.21) | 0.319 | 0.94 (0.81-1.09) | 0.395 | 0.218 |
|  | Stroke | 1.07 (0.89-1.30) | 0.463 | 0.88 (0.78-1.01) | 0.066 | 0.029 |
|  | CVD death | 0.97 (0.83-1.13) | 0.662 | 0.85 (0.74-0.96) | 0.012 | 0.149 |
| Total REM sleep time (per 5 min) | HF | 0.96 (0.93-0.99) | 0.007 | 0.97 (0.95-0.99) | 0.020 | 0.667 |
|  | MI | 1.02 (0.99-1.06) | 0.256 | 0.98 (0.95-1.02) | 0.369 | 0.088 |
|  | Stroke | 1.02 (0.97-1.08) | 0.399 | 0.96 (0.93-1.00) | 0.043 | 0.012 |
|  | CVD death | 0.99 (0.95-1.03) | 0.544 | 0.95 (0.92-0.99) | 0.005 | 0.163 |

AHI, apnea hypopnea index; CVD, cardiovascular disease; 95% CI, 95% confidence interval; HF, heart failure; HR, hazard ratio; MI, myocardial infarction; REM, rapid eye movement sleep; T90, percent time below oxygen desaturation 90%.

a Multivariable Cox regression analysis adjusted by age, race, education, marital status, smoking status, BMI, alcohol use, caffeine use, benzodiazepine use, hypertension, diabetes mellitus, triglyceride, cholesterol, HDL, sleep duration, T90 and AHI (natural log-transformed)

Supplement Table 5 Multivariable Cox regression analysis for percentage REM sleep and total REM sleep time associated with CVD events stratified by AHI **≥** 15 events/h and AHI＜15 events/h.

|  |  | AHI ≥ 15 events/h  (n=895) | | AHI＜15 events/h  (n=3597) | |  |
| --- | --- | --- | --- | --- | --- | --- |
| REM traits | CVD events | HR (95%CI) | P | HR (95%CI) | P | P_interaction_ |
| Percentage REM sleep (per 5 %) | HF | 0.85 (0.73-0.99) | 0.040 | 0.89 (0.82-0.97) | 0.005 | 0.753 |
|  | MI | 1.04 (0.84-1.27) | 0.735 | 1.01 (0.91-1.13) | 0.808 | 0.117 |
|  | Stroke | 1.09 (0.86-1.39) | 0.465 | 0.91 (0.80-1.03) | 0.117 | 0.480 |
|  | CVD death | 0.84 (0.67-1.04) | 0.101 | 0.91 (0.82-1.02) | 0.110 | 0.419 |
| Total REM sleep time (per 5 min) | HF | 0.95 (0.91-0.99) | 0.030 | 0.97 (0.95-0.99) | 0.007 | 0.729 |
|  | MI | 1.01 (0.96-1.07) | 0.677 | 1.00 (0.98-1.03) | 0.830 | 0.353 |
|  | Stroke | 1.04 (0.97-1.11) | 0.262 | 0.97 (0.94-1.00) | 0.071 | 0.580 |
|  | CVD death | 0.94 (0.88-0.99) | 0.040 | 0.97 (0.94-1.00) | 0.064 | 0.905 |

AHI, apnea hypopnea index; CVD, cardiovascular disease; 95% CI, 95% confidence interval; HF, heart failure; HR, hazard ratio; MI, myocardial infarction; REM, rapid eye movement sleep; T90, percent time below oxygen desaturation 90%.

a Multivariable Cox regression analysis adjusted by age, sex, race, education, marital status, smoking status, BMI, alcohol use, caffeine use, benzodiazepine use, hypertension, diabetes mellitus, triglyceride, cholesterol, HDL, sleep duration and T90
