# Supplementary figures and images for: Increased Rapid Eye Movement Sleep Is Associated With a Reduced Risk of Heart Failure in Middle-Aged and Older Adults
Source: Front Cardiovasc Med. 2022 Mar 29;9:771280. doi: 10.3389/fcvm.2022.771280 (PMC9001949; doi:10.3389/fcvm.2022.771280)

**Supplementary Figure 1.** Flow diagram of participant selection

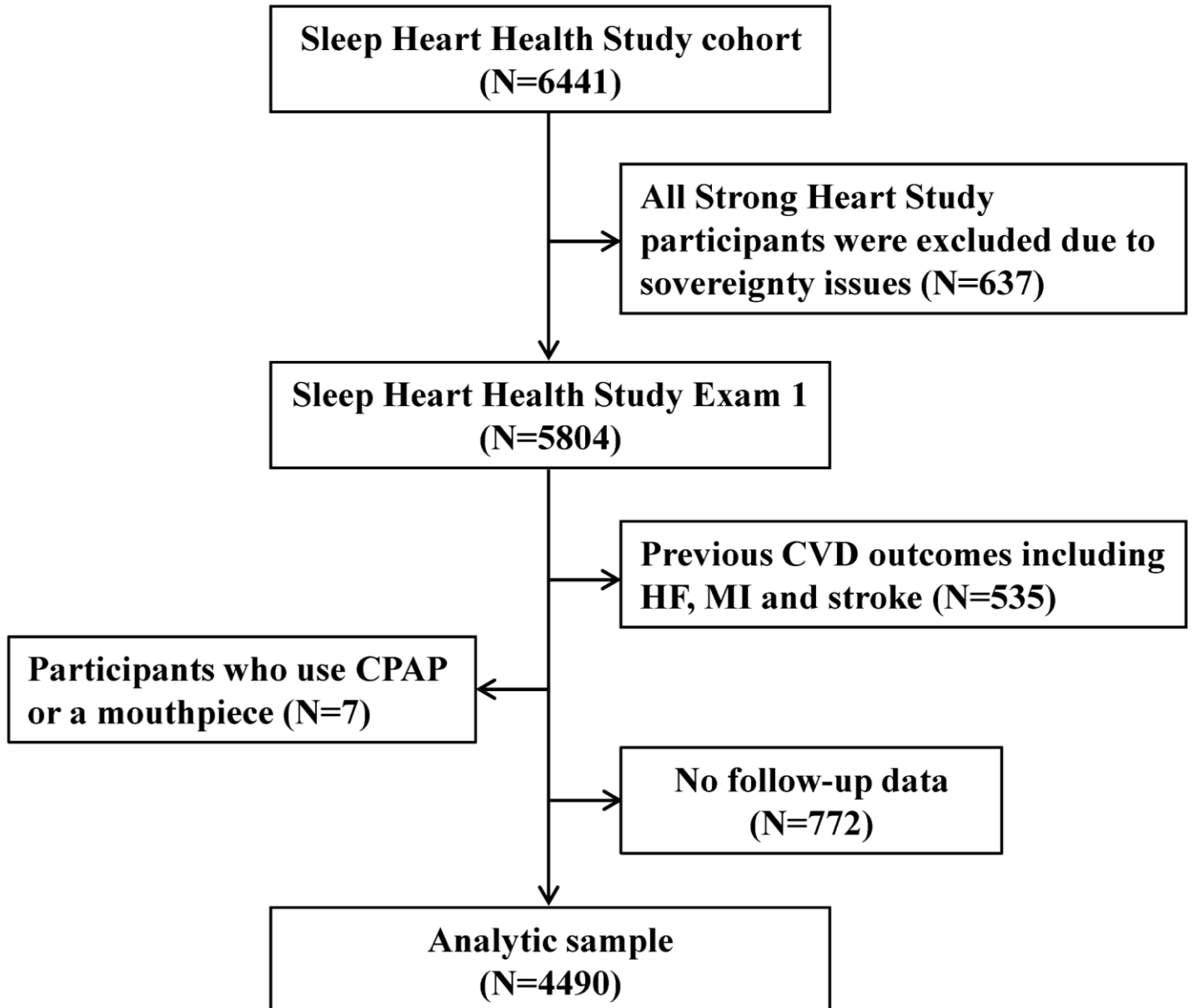

Supplement: Supplementary file 3 [file Image_1.pdf]
